# [Corrigendum] microRNA‑181a‑5p functions as an oncogene in renal cell carcinoma

**DOI:** 10.3892/mmr.2024.13182

**Published:** 2024-02-15

**Authors:** Yulin Lai, Liwen Zhao, Jia Hu, Jing Quan, Peijie Chen, Jinling Xu, Xin Guan, Yongqing Lai, Liangchao Ni

Mol Med Rep 17: 8510–8517, 2018; DOI: 10.3892/mmr.2018.8899

Following the publication of the above article and a corrigendum in 2018 that corrected details of the correspondence information for authors, errors made in Fig. 5 and the funding details (doi: 10.3892/mmr.2018.9117), an interested reader drew to the authors’ attention that, in Fig. 6 on p. 8515 showing the results of cell migration and invasion assay experiments, a pair of data panels were overlapping, such that data which were intended to show the results from differently performed experiments appeared to have been derived from the same original source. After having consulted their original data, the authors have realized that Fig. 6 was assembled incorrectly, The revised version of Fig. 6, now showing the correct data for the ‘ACHN/migratory/NC’ experiment, is shown on the next page. Note that all the authors approve of the publication of this corrigendum, and the authors are grateful to the Editor of *Molecular Medicine Reports* for granting them the opportunity to publish this. The authors regret their oversight in allowing this error to be included in the paper, and also apologize to the readership for any inconvenience caused.

## Figures and Tables

**Figure 5.**
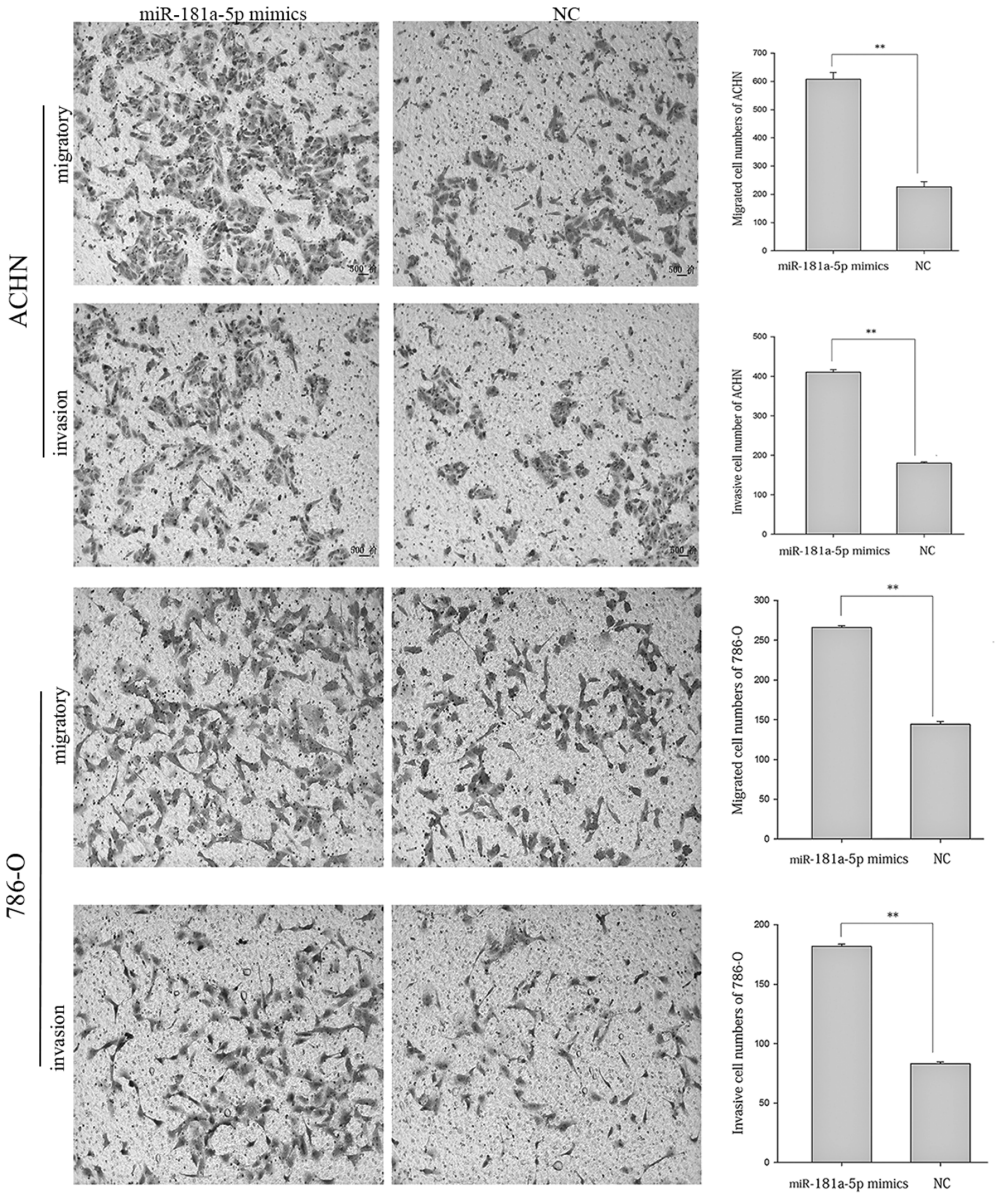
Wound scratch assay reveals the migratory ability of ACHN and 786-O cells following transfection with either miR-181a-5p mimics or NCs. Magnification, ×100. *P<0.05. miR, microRNA; NC, negative control; T, time.

